# Comparing effectiveness and safety of paclitaxel plus raltitrexed *vs.* paclitaxel alone in second-line palliative chemotherapy for metastatic gastric adenocarcinoma: A randomized phase II clinical trial

**DOI:** 10.20892/j.issn.2095-3941.2023.0112

**Published:** 2023-08-31

**Authors:** Xiaoying Zhao, Zhiyu Chen, Xiaowei Zhang, Xiaodong Zhu, Wen Zhang, Lixin Qiu, Chenchen Wang, Mingzhu Huang, Zhe Zhang, Wenhua Li, Lei Yang, Weijian Guo

**Affiliations:** 1 Department of Gastrointestinal Medical Oncology, Fudan University Shanghai Cancer Center, Shanghai 200032, China; 2 Shanghai Medical College, Fudan University, Shanghai 200032, China; 3 Department of Medical Oncology, Nantong Tumor Hospital, Nantong 226006, China

**Keywords:** Gastric adenocarcinoma, raltitrexed, paclitaxel, second-line palliative chemotherapy

## Abstract

**Objective:**

Paclitaxel (P) is a standard second-line chemotherapy in the treatment of advanced gastric cancer. This study compared the clinical outcome of a paclitaxel plus raltitrexed (RP) regimen as second-line treatment in metastatic gastric cancer (MGC) patients.

**Methods:**

An open, randomized, multi-center phase II clinical trial was conducted involving 148 patients who were randomly assigned and treated with RP [raltitrexed (3 mg/m^2^ on day 1) and paclitaxel (135 mg/m^2^ on day 1 every 3 weeks)] or P [paclitaxel (135 mg/m^2^ on day 1 every 3 weeks)] as 2^nd^-line chemotherapy. The primary endpoint was progression-free survival (PFS). The secondary endpoints were the overall response rate (ORR), overall survival (OS), and safety.

**Results:**

PFS had a tendency to be prolonged with RP compared to P (2.7 months *vs.* 1.7 months; *P* = 0.148). OS was also prolonged with RP compared to P (10.2 months *vs.* 6.1 months; *P* = 0.140). The ORR was equal in the RP and P groups (6.8% and 4.0%; *P* = 0.72). The disease control rate (DCR) in the RP and P groups was 56.2% and 36.0%, respectively. Grade 3-4 treatment-related adverse events occurred in 36.2% (RP) and 28.2% (P) of patients. Frequent grade 3-4 toxicities for RP and P were neutropenia (11.0% and 4.0%), anemia (1.4% and 4.0%), and thrombocytopenia (1.4% and 5.3%), and all grades of peripheral neurotoxicity (12.3% *vs.* 17.3%). All grades of hepatic toxicity were demonstrated for the RP and P groups based on elevated aminotransferase levels (27.4% and 14.1%). Subgroup analysis shows if MGC was combined with ascites or peritoneal involvement, the OS of the RP regimen was longer (*P* = 0.05).

**Conclusions:**

Second-line palliative chemotherapy with RP was shown to prolong the PFS and OS, especially among patients with ascites or peritoneal involvement, which warrants confirmation using larger sample studies.

## Introduction

Gastric cancer (GC) is the fifth most common malignancy worldwide. The highest incidence of GC exists in East Asian countries, especially in China and Japan^1,2^. Greater than 679,000 new GC diagnoses were recorded in China in 2015^3^. Advanced GC patients have a poor prognosis with a median survival time, if untreated, of 3-5 months. Although chemotherapy has shown a significant survival benefit, the 5-year overall survival (OS) rate of advanced GC is < 5%^4,5^. The recommended first-line chemotherapy for patients with HER2-negative GC is combination oxaliplatin or cisplatin plus 5-fluorouracil (FU) or capecitabine. The ToGA study showed that trastuzumab should be added to first-line cytotoxic therapy for HER2-positive GC^6,7^. The new standard of care is chemotherapy combined with immunotherapy. Among the gastric cancer population with a PD-L1 combined positive score (CPS) ≥ 5 in CheckMate 649, the 2-year OS of nivolumab combined with chemotherapy was 39%, while the 2-year OS in the chemotherapy alone group was 15%.

In the second-line therapy setting, ramucirumab was the only molecular-targeted drug in a global phase III clinical trial^8^. Single docetaxel, irinotecan, and paclitaxel significantly prolong OS compared with best supportive care (BSC). Previous clinical studies involving second-line combination chemotherapy have not shown improved efficacy^9,10^.

Thymidylate synthase is a well-established target enzyme for GC therapy. The mechanism underlying 5FU resistance has been investigated with a focus on the level of the thymidylate synthase (TS) ternary complex formed with fluoro-deoxyuridine monophosphate (FdUMP)^11–13^. Raltitrexed is a specific TS inhibitor that does not require modulation effects on RNA^14–16^. A meta-analysis that included 11 studies with 4622 colorectal cancer (CRC) patients reported equivalent OS and overall response rates (ORRs) with acceptable toxicities between traditional 5FU- and raltitrexed-based regimens^16–19^. Indeed, no cross-resistance between raltitrexed and 5FU was reported^20–24^. This study was designed to compare the efficacy and safety of second-line palliative chemotherapy with paclitaxel plus raltitrexed and paclitaxel alone in patients with metastatic gastric adenocarcinoma (MGC) who had not received 1^st^-line 5FU treatment.

## Materials and methods

### Patient screening and stratification

The patient inclusion criteria were as follows: age ≤ 18 years; histologically-proven gastric or esophagogastric junction adenocarcinoma; measurable and/or assessable metastatic disease according to RECIST 1.0 criteria, or locally recurrent disease associated with one or more measurable lymph nodes; Eastern Cooperative Oncology Group (ECOG) performance status > 2; progression after 1^st^-line 5FU treatment with oxaliplatin plus capecitabine (XELOX) or folinic acid, FU, and oxaliplatin [FOLFOX (with addition of trastuzumab for HER2-positivity)]; ≥ 6 weeks from prior radiotherapy and ≥ 3 weeks from surgery; and adequate hepatic, renal, and hematologic function. Similarly, the exclusion criteria were as follows: concurrent cancer; neuropathy; brain or leptomeningeal involvement; uncontrolled significant co-morbid conditions; or if patient could not comprehend the purpose of the study and could not comply with study requirements. The study was conducted in full accordance with the International Conference on Harmonization Good Clinical Practice guidelines and the Declaration of Helsinki and was approved by the Ethics Committee of the provincial government of Innsbruck in August 2012. This study was approved by the Ethics Committee of Fudan University Cancer Center (Approval No. 1309127-10). All the participants were provided written informed consent before enrolment and commencement of the study.

### Treatment

This was a randomized, multicenter, open-label, phase II clinical study in patients with histologically-proven, inoperable, locally advanced or MGC. Patients were randomly assigned (1:1) to receive raltitrexed (3 mg/m^2^ on day 1) and paclitaxel (135 mg/m^2^ on day 1 every 3 weeks) (RP) or paclitaxel 135 mg/m^2^ on day 1 every 3 weeks) (P) as 2^nd^-line palliative chemotherapy. The dose modification criteria were predefined. Treatment continued until disease progression, unacceptable toxicity, and death or consent to withdraw. Treatment can be continued up to 10 cycles or discontinued earlier at the discretion of the principal investigator.

### Evaluation and outcomes

A complete medical history was obtained and a physical examination was performed before randomization, including a complete blood count (CBC), blood chemistries, and tumor assessments. Tumor measurements were obtained every 6 weeks until progression was demonstrated in both arms, as assessed by Response Evaluation Criteria in Solid Tumors (RECIST) 1.0 criteria. Progression-free survival (PFS) was measured from the date of randomization to radiographically-documented progressive disease (PD) or death due to any cause. The OS was measured from the date of randomization-to-date of death from any cause. Toxicities were graded according to the National Cancer Institute of Canada Common Toxicity Criteria (version 3.0). Quality of life was assessed at the same time as tumor assessments and data were collected every 3 months after disease progression using the European Organization for Research and Treatment of Cancer Quality of Life Questionnaire (QLQ)-C30 (version 3).

### Statistical analysis

The primary endpoint (PFS) and secondary endpoints (OS, ORR, and safety) were determined and analyzed. The Kaplan-Meier statistical method was used to calculate the PFS and OS. ORRs were compared using a χ^2^ test. PFS and OS were calculated on the basis of the predefined full analysis population (all randomly assigned and treated patients). Patients were considered assessable for response if the patients received two or more chemotherapy cycles. Safety analyses were included for all treated patients and involved an analysis of treatment based on adverse events, including events possibly or probably related to the study medication regardless of causality.

## Results

### Patients

A total of 148 patients [raltitrexed plus paclitaxel (RP) group, *n* = 73; paclitaxel (P) group, *n* = 75] were randomly assigned to treatment regimen between August 2014 and December 2017. All patients received the medication according to protocol and the outcome was analyzed for efficacy and safety. Ninety-four patients were male and fifty-four patients were female. Greater than 90% of patients had an ECOG score of 1. Both treatment groups were well-balanced with respect to baseline characteristics (**Table 1**).

**Table 1:** Patient and cancer baseline characteristics

Characteristic	Treatment (No. of patients)					
	RP (*n* = 73)		P (*n* = 75)		Total (*n* = 148)	
	No.	%	No.	%	No.	%
Gender						
Male	48	66.0	46	61.3	87	63.5
Age, years						
Median	56.2		53.5		55.3	
Range	25–71		27–74		25–74	
< 60	63	86.3	59	78.7	122	82.4
≥ 60	10	13.7	16	11.3	26	17.6
ECOG						
0	1	1.4	2	2.7	3	2.0
1	68	93.2	69	92.0	137	92.6
2	4	6.4	4	5.4	8	5.4
Primary tumor site						
GE junction	3	4.1	3	4.0	6	4.1
Fundus	5	6.8	6	8.0	11	7.4
Antrum	55	75.3	49	65.3	104	70.2
Body	10	13.7	17	22.7	27	18.2
No. of organs involved						
1	2	2.7	1	1.3	3	2.0
2	7	9.6	4	5.3	11	7.4
> 2	65	89.0	70	93.3	135	91.2
Prior therapy						
XELOX	45	61.7	49	65.3	94	63.5
SOX	13	17.8	12	16.0	25	16.9
ECF-like	13	17.8	13	17.3	26	17.6
Other	2	2.7	1	1.3	3	2.0

RP, raltitrexed plus paclitaxel; P, paclitaxel.

### Treatment

The median duration of therapy was 3.5 cycles for RP (range, 1-15 cycles) and 4 cycles for P (range, 1-12 cycles). Dose reductions occurred in 12 patients in the RP group (16.4%) and 8 patients in the P group (10.7%). Neutropenia and thrombocytopenia were the most prominent adverse events leading to cycle delay and dose reduction in the RP and P groups. The most common adverse event leading to dose reduction in the RP group was neutropenia. The main reason for therapy discontinuation was PD in both groups.

### Efficacy: primary end points (OS and PFS)

**Figure 1:**
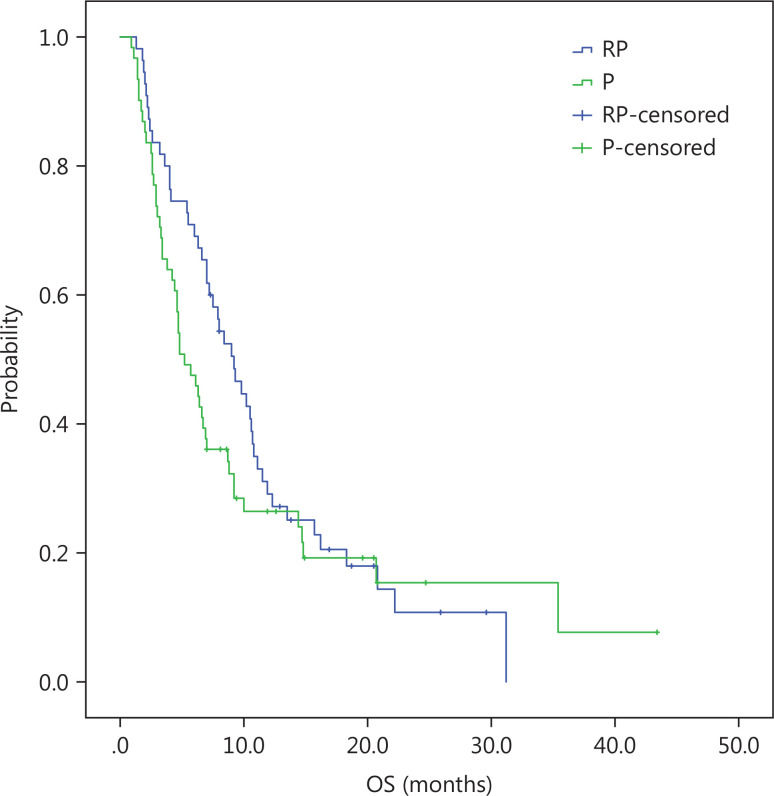
ITT, Kaplan-Meier estimates of time to overall survival among advanced gastric cancer patients treated with RP or P.

**Figure 2:**
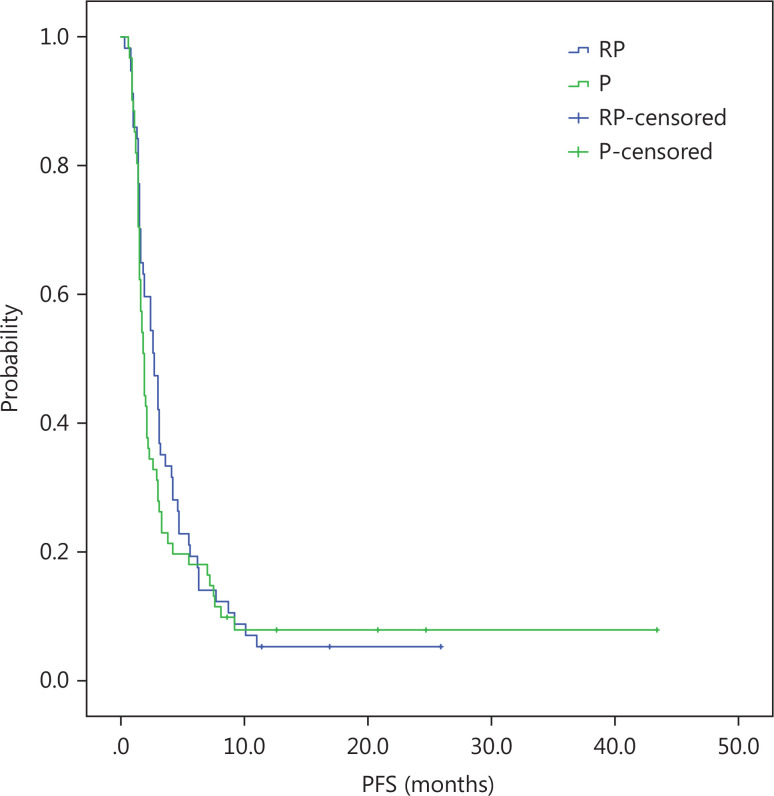
ITT, Kaplan-Meier estimates of time to progression survival among advanced gastric cancer patients treated with RP or P.

**Figure 3:**
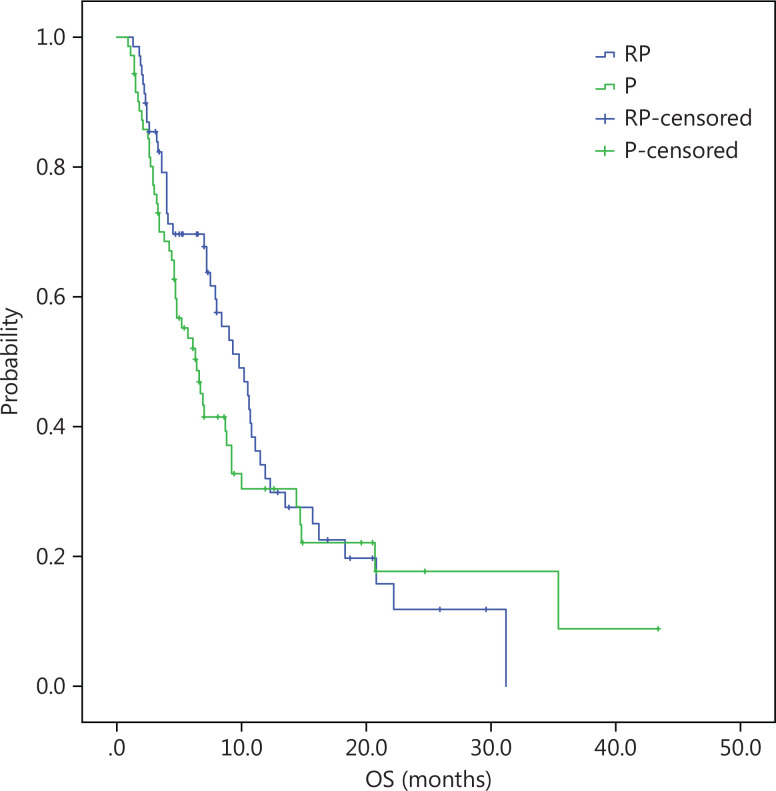
PP, Kaplan-Meier estimates of time to overall survival among advanced gastric cancer patients treated with RP or P.

**Figure 4:**
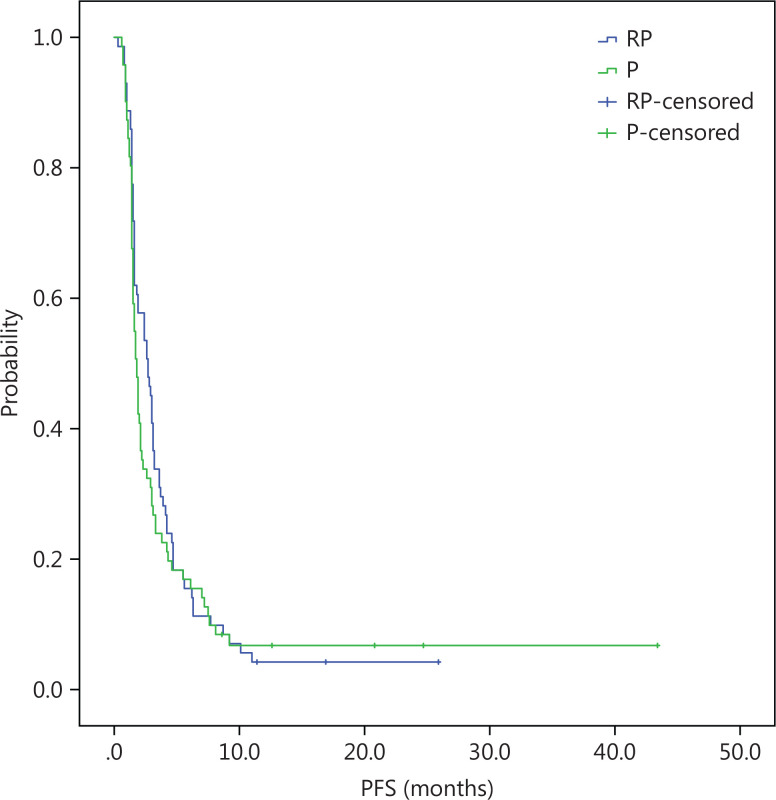
PP, Kaplan-Meier estimates of time to progression survival among advanced gastric cancer patients treated with RP or P.

At a median follow-up duration of 13 months, the median OS was longer in the intention-to-treat (ITT) population of the RP group than the P group (10.2 months, 95% CI = 8.2-12.2 *vs.* 6.1 months, 95% CI = 4.4-7.8; log-rank *P* = 0.14). There was a trend towards longer median OS with RP than P, but did not reach clinical significance (**Figure 1**). The PFS was similar in the RP and P groups (2.7 months, 95% CI = 2.1-3.8 *vs.* 1.7 months, 95% CI = 1.4-2.0; log-rank *P* = 0.148; **Figure 2**). Similar to the ITT population, there was a trend towards a longer median OS with RP than P in the per protocol (PP) population (10.8 months, 95% CI = 9.5-12.1 *vs.* 6.9 months, 95% CI = 4.2-9.6; log-rank *P* = 0.21; **Figure 3**) with a similar PFS (3.0 months, 95% CI = 2.4-3.5 *vs.* 1.9 months, 95% CI = 1.6-2.2; log-rank *P* = 0.22; **Figure 4**).

We did not detect significant differences between different tumor sites and prior chemotherapy; however, in patients with more than two organs involved, the PFS was slightly longer in the RP group than the P group (2.8 months, 95% CI = 2.2-3.5 *vs.* 1.9 months, 95% CI =1.6-2.3; log-rank *P* = 0.09), but with no statistical differences.

### Efficacy: secondary end point (ORR)

The ORR was equal for the RP (6.8%) and XELOX groups (4.0%) (*P* = 0.72; **Table 2**). One patient achieved a complete response (CR) in the P group. The partial response rate (PRR) was 6.8% in the RP group and 2.7% in the P group. The DCR was 56.2% in the RP group and 36% in the P group.

**Table 2:** Best overall response rate

Parameter	Treatment (No. of patients)			
	RP (*n* = 73)		P (*n* = 75)	
	No.	%	No.	%
Overall response rate	5	6.8	3	4.0
Complete response	0	0.0	1	1.3
Partial response	5	6.8	2	2.7
No change/stable disease	36	49.3	24	32.0
Progressive disease	32	43.8	48	64.0
Disease control rate	41	56.2	27	36.0

RP, raltitrexed plus paclitaxel; P, paclitaxel.

### Safety and Quality of life (QOL): secondary end point

Grade 3-4 treatment-related adverse events occurred in 60.6% and 57.5% of patients in the RP and P groups, respectively. Frequent grade 3-4 toxicities in the RP and P groups were neutropenia (11% *vs.* 4%), anemia (1.4% *vs.* 4%), thrombocytopenia (1.4% *vs.* 5.3%), and all grades of peripheral neurotoxicity (1.4% *vs.* 2.7%); no febrile granulocyte deficiency occurred in either group. All grades of hepatic toxicity were reported in the RP and P groups based on elevated aminotransferase levels (27.4% *vs.* 14.1%). The major treatment-based adverse events are summarized in **Table 3**. Due to the short treatment cycle, patients often only completed the QOL questionnaire 1-2 times, thus the results could not be compared. Analysis of baseline characteristics and the OS showed that the RP regimen was more favorable for poorly differentiated or undifferentiated pathologic types (*P* = 0.09). Among MGC patients with ascites, peritoneal involvement, or > 2 metastasis sites, the RP regimen was shown to be more beneficial (*P* = 0.05).

**Table 3:** Major treatment-based adverse events

Parameter	Treatment (No. of patients)	
	RP (*n* = 73) %	P (*n* = 75) %
Grade 3-4 adverse events	36.2	28.2
Grade 3-4 neutropenia	11.0	4.0
Grade 3-4 anemia	1.4	4.0
Grade 3-4 thrombocytopenia	1.4	5.3
All grades of peripheral neurotoxicity	12.3	17.3
All grades of hepatic toxicity based on elevated aminotransferase levels	27.4	14.1

RP, raltitrexed plus paclitaxel; P, paclitaxel.

## Discussion

This study was initiated in 2014. Second-line standard treatment is still single-agent chemotherapy and 3^rd^-line immunotherapy is not approved for subsequent treatment. The RP and P groups did not receive immunotherapy as subsequent treatment. P is used universally as 2^nd^-line chemotherapy in the treatment of GC. Ramucirumab is an antagonist of vascular endothelial growth factor receptor 2 (VEGFR2), and the combination with P further improves the therapeutic effect; however, ramucirumab is not currently available in China. Previous combined chemotherapies as 2^nd^-line treatments have not been successful, although clinical studies have been reported. Previous phase II clinical studies involving combined irinotecan and cisplatin or S-1 have failed, which may be because the platinum or FU drugs have failed as 1^st^-line treatment. There is no cross-resistance between raltitrexed and FU. The current study was designed to compare the efficacy and safety of 2^nd^-line palliative chemotherapy with RP and P alone in patients with MGC who progressed after 1^st^-line FU treatment.

In the present study the combination dose of P was adjusted to 240 mg/m^2^ with a dosing frequency and interval of every 3 weeks on days 1, 8, and 15 to meet the primary endpoint (PFS). The adjusted dose for treatment was well-tolerated, despite the higher cumulative P dose with shorter infusion schedules (30 min *vs.* 3 h) delivered without premedication for unselected patients with MGC. In the current study we chose P (135 mg/m^2^) and raltitrexed (3 mg/m^2^) as the study group to determine whether the combination regimen was superior to a single-drug (P) regimen.

As expected, the ORR was low in each group, but the DCR was 56.2% and 36% in the combination and P only groups, respectively. Although the OS and PFS were similar in the ITT and PP populations, the absolute increased OS time in the ITT population was 4.1 months. Subgroup analysis also suggested that the RP regimen was favorable (*P* = 0.09) if the pathologic type was poorly differentiated or undifferentiated, and if the patients had ascites, peritoneal involvement, or > 2 metastatic sites (*P* = 0.05).

The hematologic toxicity profile in the RP and P group was similar. The incidence of hepatotoxicity was higher in the RP group because of the combination of raltitrexed and P.

The survival time in the monotherapy group was shorter than 3-4 months, as reported in the existing literature, likely because P was administered once every 3 weeks in the current study. A clinical study in which P-albumin was administered as 2^nd^-line treatment every 3 weeks in patients with GC also showed worse efficacy than when administered weekly. Another reason for this finding was that the P dosage used in the current study had a lower dose in the 3-week standard dosage regimen, considering the comparability between the experimental and the single-drug groups. The application of low-dose P in China conforms to the body habitus of Chinese patients. Even though lower doses of P were chosen in this study, hematologic toxicity was observed. Fortunately, the addition of an antineoplastic agent in the combination group did not significantly increase the hematologic toxicity and was well-tolerated. At the time of this study, there was no standard 3^rd^-line treatment for advanced gastric cancer. The 2^nd^-line PFS was relatively short and exhibited rapid progression. Therefore, the vast majority of patients did not undergo subsequent treatment.

## Conclusions

It is our opinion that appropriate dosage and usage of P and antimetabolites may have a greater role in 2^nd^-line therapy of advanced GC. The P combined with raltitrexed group had improved efficacy with favorable tolerance and good safety, especially in the individual sub-groups. It is therefore warranted to conduct a randomized controlled study for this subgroup in the future.

## Data Availability

The authors confirm that the data supporting the findings of this study are available within the article.

## References

[1] Ajani JA, Lee J, Sano T, Janjigian YY, Fan D, Song S (2017). Gastric adenocarcinoma. Nat Rev Dis Primers.

[2] Balakrishnan M, George R, Sharma A, Graham DY (2017). Changing trends in stomach cancer throughout the world. Curr Gastroenterol Rep.

[3] Nie Y, Wu K, Yu J, Liang Q, Cai X, Shang Y (2017). A global burden of gastric cancer: the major impact of China. Expert Rev Gastroenterol Hepatol.

[4] Lau CH, Wu X, Chung VC, Liu X, Hui EP, Cramer H (2016). Acupuncture and related therapies for symptom management in palliative cancer care: systematic review and meta-analysis. Medicine (Baltimore).

[5] Zhu XD, Huang MZ, Wang YS, Feng WJ, Chen ZY, He YF (2022). XELOX doublet regimen *vs.* EOX triplet regimen as first-line treatment for advanced gastric cancer: an open-labeled, multicenter, randomized, prospective phase III trial (EXELOX). Cancer Commun.

[6] Van Cutsem E, Sagaert X, Topal B, Haustermans K, Prenen H (2016). Gastric cancer. Lancet.

[7] Wong NACS, Amary F, Butler R, Byers R, Gonzalez D, Haynes HR (2018). HER2 testing of gastro-oesophageal adenocarcinoma: acommentary and guidance document from the Association of Clinical Pathologists Molecular Pathology and Diagnostics Committee. J Clin Pathol.

[8] Fuchs CS, Tomasek J, Yong CJ, Dumitru F, Passalacqua R, Goswami C, REGARD Trial Investigators (2014). Ramucirumab monotherapy for previously treated advanced gastric or gastro-oesophageal junction adenocarcinoma (REGARD): an international, randomised, multicentre, placebo-controlled, phase 3 trial. Lancet.

[9] Ilson DH (2017). Advances in the treatment of gastric cancer. Curr Opin Gastroenterol.

[10] Mizra Kaya D, Harada K, Shimodaira Y, Amlashi FG, Lin Q, Ajani JA (2017). Advanced gastric adenocarcinoma: optimizing therapy options. Expert Rev Clin Pharmacol.

[11] Wang W, McLeod HL, Cassidy J, Collie-Duguid ES (2007). Mechanisms of acquired chemoresistance to 5-fluorouracil and tomudex: thymidylate synthase dependent and independent networks. Cancer Chemother Pharmacol.

[12] Chen XD, He FQ, Chen M, Tang LC, Tang XL (2016). Can S-1 replace fluorouracil for advanced gastric cancer? A PRISMA-compliant systematic review and meta-analysis. Medicine (Baltimore).

[13] Ajani J (2006). Review of capecitabine as oral treatment of gastric, gastroesophageal, and esophageal cancers. Cancer.

[14] Massacesi C, Terrazzino S, Marcucci F, Rocchi MB, Lippe P, Bisonni R (2006). Uridine diphosphate glucuronosyl transferase 1A1 promoter polymorphism predicts the risk of gastrointestinal toxicity and fatigue induced by irinotecan-based chemotherapy. Cancer.

[15] Scheithauer W, Kornek GV, Ulrich-Pur H, Penz M, Raderer M, Salek T (2001). Oxaliplatin plus raltitrexed in patients with advanced colorectal carcinoma: results of a Phase I-II trial. Cancer.

[16] Raderer M, Fiebiger W, Wrba F, Scheithauer W (2000). Fatal liver failure after the administration of raltitrexed for cancer chemotherapy: a report of two cases. Cancer.

[17] Barni S, Ghidini A, Coinu A, Borgonovo K, Petrelli F (2014). A systematic review of raltitrexed-based first-line chemotherapy in advanced colorectal cancer. Anticancer Drugs.

[18] Decoster L, Neyns B, Akouaouach H, Fontaine C, Schallier D, De Grève JL (2004). Efficacy of infusional biomodulated 5-fluorouracil in metastatic colorectal cancer after raltitrexed failure. Anticancer Res.

[19] Vaflard P, Ederhy S, Torregrosa C, André T, Cohen R, Lopez-Trabada D (2018). Fluoropyrimidines cardiac toxicity: 5-fluorouracil, capecitabine, compound S-1 and trifluridine/tipiracil. Bull Cancer.

[20] Papanastasopoulos P, Stebbing J (2014). Molecular basis of 5-fluorouracil-related toxicity: lessons from clinical practice. Anticancer Res.

[21] Shao G, Liu R, Ding W, Lu L, Li W, Cao H (2018). Efficacy and safety of raltitrexed-based transarterial chemoembolization for colorectal cancer liver metastases. Anticancer Drugs.

[22] El-Mesallamy HO, El Magdoub HM, Chapman JM, Hamdy NM, Schaalan MF, Hammad LN (2018). Biomolecular study of human thymidylate synthase conformer-selective inhibitors: new chemotherapeutic approach. PLoS One.

[23] Mori R, Futamura M, Tanahashi T, Tanaka Y, Matsuhashi N, Yamaguchi K (2017). 5FU resistance caused by reduced fluoro-deoxyuridine monophosphate and its reversal using deoxyuridine. Oncol Lett.

[24] Huang M, Yang Y, Zhu X, Chen Z, Zhang W, Wang C (2021). A prospective phase II study of raltitrexed combined with S-1 as salvage treatment for patients with refractory metastatic colorectal cancer. Asia Pac J Clin Oncol.

